# Bioengineered Cellular and Acellular Therapies for Ischemic Heart Disease in Clinically Relevant Models

**DOI:** 10.3390/bioengineering13010081

**Published:** 2026-01-12

**Authors:** Kelsey C. Muir, Clark Zheng, Keertana Yalamanchili, Riya Reddy, Alexander Joseph, Jad Hamze, Dwight D. Harris, Frank W. Sellke

**Affiliations:** 1 Department of Surgery, Division of Cardiothoracic Surgery, Warren Alpert Medical School, Providence, RI 02903, USA; 2 Cardiovascular Research Center, Department of Surgery, Rhode Island Hospital, Providence, RI 02903, USA

**Keywords:** ischemic heart disease, stem cells, extracellular vesicles, extracellular matrix, regenerative therapy, angiogenesis, large animal models

## Abstract

Despite significant improvements in revascularization strategies and medical management, ischemic heart disease (IHD) remains the top cause of mortality and disability worldwide. The myocardium lacks regenerative capacity and consequently, recovery depends on re-establishing microvascular integrity and sustaining angiogenesis to preserve viable myocardium. Emerging and novel bioengineering approaches, such as stem cells, extracellular vesicles (EVs), and matrix-based strategies, seek to address this unmet need by promoting neovascularization and structural restoration. However, clinical translation remains limited by poor engraftment, product variability, and arrhythmogenic risk. Large animal models provide a clinically relevant platform to thoroughly investigate these interventions and ideally enhance their translational potential. This review discusses cellular approaches leveraging stem and progenitor cells and acellular modalities using extracellular vesicles, growth factors, or extracellular matrix-based scaffolds with an emphasis on large animal translational models and clinical trials.

## 1. Introduction

Ischemic heart disease (IHD) remains a leading cause of death and disability globally, accounting for nearly nine million annual deaths, and imposing substantial patient and socioeconomic burden [1]. Myocardial infarction (MI), the most severe acute manifestation of IHD, results in abrupt loss of blood flow leading to cardiomyocyte death and progressive ventricular remodeling [2]. Despite vast resources and advancements in percutaneous and surgical revascularization strategies as well as guideline-directed medical therapy (GDMT), a significant number of patients experience adverse ventricular remodeling that progresses into heart failure [3]. Essentially, the ischemic territory contracts uncoordinated and weakly compared to the rest of the heart, causing ventricular dilation, mitral regurgitation, and a reduction in ejection fraction [4]. Given the limited innate regenerative ability of cardiomyocytes, this myocardial injury triggers permanent fibrotic scar formation and ultimately results in the loss of cardiac function [5].

Given these constraints and the known limitations of current optimized therapeutic strategies, an urgent need exists for therapies with capabilities to rebuild microvascular networks, restore myocardial structure, and promote durable functional recovery. To this end, recent advances in cellular and acellular therapies have become a promising frontier. Cellular approaches include stem and progenitor cells which aim to enhance neovascularization and restore contractile units, while acellular therapies, such as extracellular vesicles (EVs) and extracellular matrix (ECM) biomaterials leverage paracrine signaling and provide structural scaffolding to promote tissue repair [6]. While early preclinical work showed promise, translation into meaningful clinical therapies has remained slow due to inconsistent cell retention, variability in product, safety concerns regarding arrhythmogenic potential, immune responses, and difficulties in delivery methods [7]. Ideally, to bridge this gap, large animal models offer a potential solution in that they closely mirror human cardiac anatomy, physiology, and electrophysiology, allowing a more rigorous assessment of these therapies.

In this review, recent advances in cellular and acellular (EVs and ECM-based) regenerative approaches are detailed in regard to IHD treatment, while emphasizing large animal translational studies and human clinical trials.

## 2. Large Animal Models as Clinically Relevant Platforms

Large-animal systems are key for connecting cardiac research with clinical translation. Among these, the porcine heart offers a uniquely faithful analogue to human coronary physiology, myocardial metabolism, and chamber mechanics. Unlike rodents, whose rapid heart rate and altered excitation-contraction coupling constrain translational interpretation, swine exhibit comparable heart size, coronary distribution, and hemodynamic load, allowing human-scale assessment of ischemic pathophysiology and response to bioengineered therapies [8,9]. In particular, surgically induced chronic ischemia models, including the ameroid constrictor and coronary microembolization systems, have become cornerstones for evaluating regenerative and angiogenic interventions [10].

### 2.1. Large Animal Models of Chronic Ischemia and Infarction

The ameroid constrictor technique remains the benchmark for establishing progressive, regionally confined ischemia in the porcine heart. First described for cardiovascular research in the mid-twentieth century and later refined for translational bioengineering, this model uses a stainless-steel ring containing a hygroscopic casein insert placed around a proximal epicardial coronary artery, most commonly the left circumflex coronary artery (LCx) [11]. As the insert gradually swells, luminal occlusion occurs over approximately three to four weeks, generating reproducible, chronic hypoperfusion that stimulates collateral formation while preserving animal survival [12]. The resulting pathophysiology reproduces clinical features of human chronic coronary disease, including microvascular dysfunction, impaired vasoreactivity, and collateral-dependent perfusion [12,13,14].

The versatility of the porcine ischemia platform extends beyond the ameroid constrictor to include microembolization-based approaches that reproduce the diffuse microvascular disease often seen in patients with diabetes, obesity, and metabolic syndrome. The microembolization model employs selective intracoronary infusion of calibrated microspheres to achieve distal arteriolar obstruction, producing patchy ischemia and contractile dysfunction without acute infarction [15]. This design allows investigation of microvascular integrity, hibernating myocardium, and the cellular adaptations that accompany impaired perfusion of the inner myocardial layer. Unlike the focal, collateral-driven pattern of the ameroid system, microembolization generates a spatially heterogeneous injury field suitable for assessing diffuse microvascular remodeling, fibrosis, and chronic inflammation, which align with the clinical presentation of microvascular angina and heart failure with preserved ejection fraction [15].

Building on these foundational models, recent iterations developed in translational bioengineering laboratories have added refinements to incorporate comorbid metabolic stress, sex differences, and advanced imaging-guided sampling, further increasing reproducibility and survival [15]. For instance, controlled surgical placement of ameroid constrictors on the left circumflex artery allows predictable ischemic territory formation, while incorporation of myocardial perfusion mapping enables longitudinal assessment of collateral development [16]. These refinements have supported mechanistic studies of endothelial adaptation, mitochondrial signaling, and angiogenic therapy in the context of chronic ischemia. Specifically, studies involving extracellular vesicle administration demonstrated measurable improvements in perfusion and capillary density associated with the downregulation of anti-angiogenic mediators and the restoration of endothelial homeostasis [17]. Parallel studies integrating proteomic and metabolomic analyses revealed that the ischemic myocardium undergoes coordinated metabolic reprogramming toward oxidative resilience when subjected to specific pharmacologic interventions, reinforcing the translational fidelity of this model [18]. Collectively, these advancements confirm that the porcine ameroid constrictor and microembolization models not only reproduce the structural and physiological hallmarks of human ischemic heart disease but also support high-resolution assessment of molecular and functional therapeutic endpoints.

Beyond porcine systems, ovine models have also been developed to study myocardial remodeling, heart failure, and surgical interventions. Sheep exhibit cardiovascular anatomy and physiology that are closer to humans than small species, including comparable heart rates, intracardiac pressures, and heart-to-body weight ratios, making them valuable for translational cardiac research [19]. They have also been shown to tolerate cardiopulmonary bypass and repeated thoracotomies well, allowing longitudinal studies of ventricular remodeling and chronic ischemia [19]. In addition, Duchenne et al. demonstrated that the sheep model supports controlled regional myocardial workload and reproducible patterns of regional remodeling, validating its suitability for evaluating contractility and mechanical adaptation after ischemic injury [20]. Overall, studies have shown that both swine and sheep reproduce the key structural and functional hallmarks of human left-ventricular dysfunction, providing complementary systems for translational testing [21].

Historically, canine models have also played a central role in defining mechanisms of myocardial infarction, collateral vessel growth, and ischemic preconditioning. However, dog hearts possess extensive native coronary collaterals, a feature that facilitated early mechanistic studies but also introduced variability in infarct size and chronic remodeling outcomes [22]. In addition, although canine models were essential in establishing the sequence from infarction to heart failure, they are now rarely used due to ethical and regulatory constraints. Consequently, contemporary large-animal research has shifted toward swine and sheep, which provide more reproducible infarct territories and closer anatomic correlation to human coronary circulation [21,22].

### 2.2. Advantages over Small Animal Models

Rodent models remain essential for understanding molecular mechanisms of ischemic injury; however, fundamental differences between rodents and humans, both in terms of cardiac physiology and scale, limit their translational capacity. The rodent myocardium exhibits a markedly higher basal metabolic rate, a higher heart rate, and distinct excitation-contraction coupling properties that complicate direct extrapolation to human systems [9]. Moreover, the coronary anatomy of rodents differs substantially from that of humans: their native collateral network is limited, and coronary branching is simpler, lacking the hierarchical structure seen in human hearts [23,24]. Consequently, assessments of regional perfusion, ventricular mechanics, or catheter-based interventions in rodents do not fully replicate the hemodynamic complexity of human ischemic heart disease.

**Figure 1:**
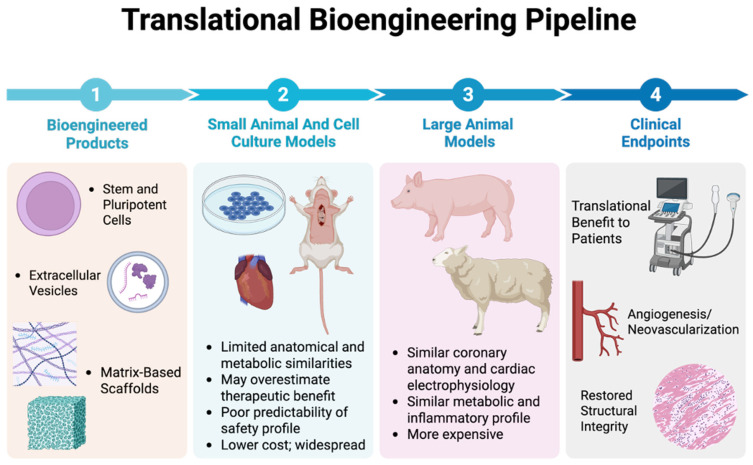
**Translational Bioengineering Pipeline.** Graphical overview of the translational continuum from bioengineered therapeutic design to clinical endpoints in ischemic heart disease. ***Step 1:*** Bioengineered cellular and acellular products, such as stem or pluripotent cells, extracellular vesicles, and matrix-based scaffolds, which represent emerging therapeutic strategies for myocardial repair. ***Step 2:*** Cell culture and small-animal models provide mechanistic insight; however, exhibit limited anatomical and metabolic fidelity, may overestimate efficacy, and often lack predictive safety relevance. ***Step 3:*** Large-animal models, including swine, more closely replicate human coronary anatomy, cardiac electrophysiology, and metabolic responses, enabling realistic safety profiling, reliable perfusion and angiogenic analysis, and cellular pathway responses. ***Step 4:*** Translational outcomes, including neovascularization and structural restoration, represent clinically meaningful endpoints that bridge experimental bioengineering with patient benefit. This pipeline emphasizes the role of large-animal models as a crucial intermediary for advancing bioengineered cellular and acellular therapies toward clinical application in IHD.

Other small-animal systems, including rabbits and guinea pigs, have been widely used to study myocardial infarction and ischemia–reperfusion through coronary artery ligation or cryoinjury, providing intermediate complexity between murine and large-animal models [25,26]. Zebrafish have also advanced cardiac research through models of myocardial regeneration, enabling investigation of intrinsic repair mechanisms and genetic regulators of myocardial recovery [27]. Although these systems yield valuable insights into molecular signaling and regenerative biology, they remain limited in reproducing the physiological scale, hemodynamic load, and procedural environment of human ischemic heart disease (Figure 1).

Swine, by contrast, display near-identical coronary distribution, ventricular geometry, and myocardial oxygen consumption to humans, allowing true quantitative measurement of perfusion and contractile function [8,28]. Their body size supports implementation of human clinical instrumentation, including pressure-volume conductance catheters, intracoronary Doppler flow wires, and standard fluoroscopic or echocardiographic systems [28,29,30]. In swine, pressure-volume loop analysis enables acquisition of load-independent indices such as end-systolic elastance and diastolic compliance; by contrast, although similar methods have been applied in murine hearts, the very small chamber volumes, high heart rates and instrumentation challenges make reliable derivation of these indices more difficult in mice [31]. Furthermore, the reproducible coronary anatomy of the pig permits selective occlusion or perfusion measurement within defined vascular territories, yielding spatial correlation between physiological and molecular data.

Integration of imaging technologies within the same organism further enhances the translational relevance of large-animal models. High-field cardiac magnetic resonance imaging and positron emission tomography have been adapted for serial evaluation of perfusion, metabolism, and fibrosis in chronically ischemic swine, providing direct parallels to human diagnostic workflows [32]. Coupling these imaging modalities with myocardial biopsy or spatially resolved transcriptomic and proteomic analyses enables comprehensive mapping of ischemic adaptation at the organ, tissue, and cellular levels. For example, large-animal experiments using combined perfusion mapping and proteomics have demonstrated region-specific metabolic shifts and cytoskeletal remodeling that mirror findings in patients with chronic ischemic cardiomyopathy [17].

Together, these characteristics position large-animal systems as critical tools for bridging experimental bioengineering with clinical interventions. By replicating human-scale anatomy, coronary architecture, and procedural conditions, swine models enable quantitative, mechanistically informed testing that rodent models cannot achieve. The integration of imaging, hemodynamic, and omics analyses within the same organism also allows for a comprehensive analysis of myocardial adaptation and response to treatments over time. Overall, this convergence of physiological fidelity, procedural compatibility, and molecular resolution defines the translational value of the porcine model, supporting reliable assessment of regenerative and metabolic treatments and establishing a critical bridge to patient application (Figure 1).

## 3. Cellular Therapies

### 3.1. Defining the Scope of Cellular Therapy and Bioengineering

The utilization of cellular therapeutics, including stem cell-based regeneration and engineered platforms, has demonstrated substantial efficacy in treating degenerative diseases [33]. In the cardiac context for the treatment of IHD, there are three primary cellular modalities: mesenchymal stem cells (MSCs), induced pluripotent stem cells (iPSCs), and various cardiac progenitor cells (CPCs). The translation of these cell-based approaches relies heavily on bioengineering strategies to overcome the limitations imposed by the ischemic microenvironment [34]. Bioengineering provides the necessary solutions required to ensure adequate cell survival, functional integration, and address challenges that traditional medical treatments cannot surmount [35].

### 3.2. Stem and Progenitor Cell Delivery Modalities

#### 3.2.1. Mesenchymal Stem Cells (MSCs)

MSCs are among the most extensively studied and clinically advanced cell types investigated for IHD, owing to their favorable immunological profile, paracrine reparative capacity, and feasibility for translational application [34,35] (Table 1). MSCs are adult, multipotent stromal cells that can be isolated from several tissue sources, most commonly bone marrow, adipose tissue, and umbilical cord-derived tissues, including Wharton’s jelly. While MSCs share core phenotypic characteristics across tissue sources, such as plastic adherence, expression of CD73, CD90, and CD105, and absence of hematopoietic markers, their biological potency, proliferative capacity, and secretome composition vary substantially depending on tissue origin and donor characteristics [34,36].

Preclinical investigations of MSC therapy for IHD have been conducted across rodent, large animal, and human-derived systems, providing a hierarchical framework for translational evaluation. In small animal models, MSC therapy has been most extensively evaluated using murine and rat models of acute myocardial infarction induced by permanent or transient ligation of the left anterior descending (LAD) coronary artery. In a frequently cited rat study, bone marrow-derived MSCs from isogenic rats were intravenously administered after coronary ligation and were shown to significantly increase capillary density, reduce infarct size, and improve cardiac function [37]. Similarly, in a rabbit MI model, epicardial adipose tissue-derived MSCs were shown to improve ejection fraction, reduce infarct size and increase in vascular density [38]. Collectively, these small animal studies established the mechanistic foundation for MSC-mediated angiogenesis and cytoprotection, which subsequently motivated validation in large animal models and humans.

Transitioning toward greater clinical relevance, large animal models—most notably swine—have played a critical role in validating MSC efficacy and safety. In these studies, MSCs are typically bone marrow-derived, sourced either autologously or allogeneically, and delivered via intramyocardial injection in models of both acute and chronic myocardial ischemia [39]. Seminal work by Amado et al. and subsequent studies by Quevedo, Schuleri, and colleagues demonstrated that human or porcine bone marrow-derived MSCs improved regional contractility, reduced scar burden, and promoted reverse remodeling in swine with ischemic cardiomyopathy [40,41,42,43]. Importantly, these large animal studies highlighted that functional benefits were achieved despite minimal long-term cell engraftment, reinforcing the primary of MSC-mediated paracrine effects.

While early hypotheses suggested direct cardiomyocyte differentiation, it is now widely accepted that structural myocardial regeneration is minimal. The principal barrier to direct engraftment is the rapid clearance and death of transplanted cells within the ischemic microenvironment which severely limits their survival as well as retention [44]. Post-transplant retention is extremely low, undermining the hypothesis that therapeutic benefit arises from robust engraftment or cardiomyocyte replacement. The observed functional benefits, despite minimal cell retention, are overwhelmingly attributed to paracrine action [45]. Across experimental systems, MSCs exert their therapeutic effects predominantly through paracrine mechanisms, including secretion of pro-angiogenic (e.g., VEGF, FGF), anti-inflammatory, anti-apoptotic, and antifibrotic factors, as well as immunomodulatory cytokines and extracellular vesicles [46,47]. The most critical vectors for this paracrine signaling are EVs, including exosomes and microRNAs. These nanoscale vesicles regulate repair pathways, stimulate angiogenesis and cell proliferation, and suppress fibrosis and apoptosis [45]. Other mechanisms by which MSCs repair or rescue injured cells include the transfer of organelles (e.g., mitochondria) through tunneling nanotubes or microvesicles [47]. Furthermore, MSCs modulate macrophage polarization, suppress excessive inflammatory signaling, and enhance endothelial survival, thereby preserving microvascular integrity within the ischemic myocardium.

The encouraging results from preclinical models led to multiple human clinical trials employing both autologous and allogeneic MSCs, primarily derived from bone marrow or adipose tissue. Notably, the POSEIDON randomized trial and TAC-HFT trials evaluated transendocardial delivery of bone marrow-derived MSCs in patients with ischemic cardiomyopathy and demonstrated modest improvements in ventricular function, reduced scar size, and favorable safety profiles, with no increase in arrhythmogenic risk or immune rejection [36,48,49]. Most recently, the PREVENT-TAHA8 trial utilizing intracoronary infusions of Wharton’s Jelly MSCs showed a reduction in incidences of heart failure, readmission for heart failure, and cardiovascular mortality after an acute myocardial infarction [50]. Across clinical studies, MSC therapy has generally been well tolerated, with no consistent evidence of tumorigenicity, sustained immune sensitization, or major adverse cardiac events directly attributable to cell administration. However, efficacy outcomes have been heterogeneous, likely reflecting differences in cell source, tissue origin, dosing strategies, delivery routes, disease chronicity, and patient comorbidities. These findings underscore the importance of rigorous model selection and mechanistic alignment when extrapolating preclinical results to human populations.

#### 3.2.2. Induced Pluripotent Stem Cell (iPSC)

Induced pluripotent stem cells (iPSCs) represent a major bioengineering advance in regenerative cardiovascular medicine, offering a theoretically unlimited source of patient-specific or allogeneic cells capable of differentiating into multiple cardiac-relevant lineages. iPSCs are generated through the reprogramming of adult somatic cells, most commonly human dermal fibroblasts or peripheral blood mononuclear cells, into a pluripotent state via transient expression of defined transcription factors [51] (Table 1). This approach provides a longer lifespan, faster proliferation, and improved homogeneity compared to primary MSCs, resulting in a more scalable and reliable therapeutic cell supply [52]. This includes iPSC-derived cardiomyocytes, endothelial cells, smooth muscle cells, and mesenchymal stromal cells (iPSC-MSCs), each of which has been explored for IHD therapy.

Research using small animal models established foundational proof-of-concept for iPSC-derived cell therapy after myocardial ischemia. For example, Wendel et al. created fibrin patches seeded with human iPSC-derived cardiomyocytes (hiPSC-CMs) and grafted them onto acute infarcts in athymic rats, in which the hiPSC-CMs survived, proliferated, and patch-treated hearts demonstrated small infarct scaring and improved left ventricular fractional shortening compared to controls [53]. In another study, human iPSC-derived endothelial cells (iPSC-ECs) and iPSC-CMs were cotransplanted in mice after MI demonstrated augmentation of vascularization in the graft, promoted maturity of CMs, and improved cardiac function [54]. These rodent studies established that iPSC-derived cardiac cells can engraft and positively remodel the injured myocardium, setting the stage for larger animal experimentation. Cheng and colleagues took their investigation to this translational standard by cotransplanting iPSC-ECs and iPSC-CMs in non-human primates after ischemic reperfusion injury showing significant enhancement of graft vascularization and improved cardiac function [54].

Translation to large animal models, particularly pigs and primate models, has been crucial for evaluating therapeutic delivery, dosing, and safety at scale. Intramyocardial injection of human iPSC derivatives in infarcted pig hearts has yielded promising outcomes. In a landmark study by Ye et al., researchers generated iPSCs from adult human cells (e.g., dermal fibroblasts), differentiated them into three cardiac cell types (CMs, ECs, and smooth muscle cells), and delivered thus tri-lineage cell mixture into acute infarcted pig myocardium by intramyocardial injections combined with an insulin growth factor (IGF)-1-releasing fibrin patch [55]. The transplanted hiPSC-CMs formed new graft myocardium with organized sarcomeres, while the endothelial and smooth muscle components integrated into host vasculature. Treated swine demonstrated significantly improved LV ejection fraction, myocardial metabolism, higher arteriole density, and reduced infarct size [55]. Notably, despite the large cell dose, no ventricular arrhythmias were induced in this study, an encouraging safety signal. Different iPSC-derived cell types have been explored in swine. For example, Liao et al. compared intracardiac injections of hiPSC-MSCs versus hESC (embryonic stem cell)-derived CMs in a pig model of post-MI heart failure [56]. Notably, this study further demonstrated no difference in inducible ventricular tachyarrhythmia. At 8 weeks post-treatment, both cell types improved ejection fraction; however, only the hiPSC-MSC-treated hearts showed markedly increased vessel density with upregulated human VEGF-A, angiopoietin-1, and transforming growth factor (TGF)-α expression and reduced myocardial inflammation [56]. This suggests iPSC-MSC therapy primarily aids cardiac repair via paracrine angiogenesis and inflammation dampening, rather than direct muscle regeneration. In regard to safety profile of iPSC-derived cardiac lineages, two separate studies in both non-human primates and swine demonstrated transient but significant increase in ventricular tachycardia episodes and/or wide QRS complexes in the 2–3 weeks after treatment [57,58]. These studies highlight both the efficacy of iPSC-derived grafts and the challenges that inform human trials.

Encouraged by preclinical success, investigators have cautiously progressed to first-in-human studies of iPSC-based cardiac therapy. In Japan, which has led clinical translation of iPSC technology, a pioneering case study tested epicardial patches of hiPSC-CMs in a patient with severe ischemic cardiomyopathy (LVEF ≤ 35%) and demonstrated improved symptoms and exercise tolerance at 6 months post-treatment and PET/CT imaging showed viable graft tissue with no evidence of tumor formation [59]. The patient did not experience arrhythmias requiring intervention nor signs of immune rejection, which paved the way for an expanded phase I trial (#jRCT2053190081) to enroll additional patients. In parallel, industry-sponsored trials are moving forward. Heartseed Inc., a Japanese biotech in partnership with Novo Nordisk, developed an iPSC-derived CM spheroid product (HS-001) for direct myocardial injection. They have completed enrollment for a Phase I/II dose-escalation trial (the “LAPiS” study) delivering these iPSC-CM spheroids via intramyocardial injection during CABG surgery [60]. The first patient treated in 2023 demonstrated favorable results, but full reports are pending. Notably, the HS-001 spheroids use allogeneic hiPSC-CMs purified to remove undifferentiated cells and are derived from an iPSC line optimized for immune matching. The coming years will reveal whether the promising outcomes in animals translate to meaningful clinical improvements in patients with IHD.

While iPSC-based therapies hold great promise, several safety concerns must be addressed on the path to routine clinical use. Arrhythmogenic risk is a primary concern. The immature electrophysiological state of iPSC-CMs remains the chief barrier to safe engraftment, as poor electrical coupling can provoke serious arrhythmias [61]. Bioengineering approaches therefore prioritize maturation, using engineered heart tissues and bioreactors that combine mechanical conditioning with high-frequency electrical pacing to promote adult-like function [61]. While the risk of arrhythmia remains a critical concern, studies of stabilized cardiac patch implantation show it may not compromise electrical stability [62]. Furthermore, ongoing trials deliberately combine cell delivery with cardiac surgery, so patients can be supported and monitored closely during the vulnerable early period. Tumorigenicity is the second major concern. Pluripotent stem cells can form teratomas if undifferentiated cells are transplanted, so thorough purification and validation of iPSC-derived products are essential. Preclinical models and the first human cases have so far shown no evidence of tumor formation; however, to bolster safety, researchers have built fail-safes such as suicide genes engineered into iPSCs to eliminate any proliferating off-target cells [63]. Immune rejection is also a key issue, particularly for allogeneic iPSC therapies. Current trials use allogeneic cells from iPSC lines that are selected for immune compatibility (e.g., homozygous HLA donors) and/or rely on immunosuppressive drugs.

#### 3.2.3. Cardiac Progenitor Cells (CPCs) and Cardiosphere-Derived Cells (CDCs)

Resident CPCs were initially seen as a source for endogenous cardiac repair, possessing the ability to differentiate into multiple cardiovascular cell types [64] (Table 1). The tyrosine kinase protein CD117, referred to as cKit+, was introduced as a bona fide marker to identify and study these cells in the adult heart. Subsequent work has expanded the field to include multiple resident cardiac progenitor subsets, such as cardiosphere-derived cells (CDCs), stem cell antigen 1 (Sca-1+) cells, side-population cells, and developmental progenitors (lsl1+, Nkx2.5+), shifting emphasis from direct cardiomyocyte replacement toward paracrine-mediated repair, angiogenesis, and modulation of post-ischemic remodeling [65].

Numerous small animal studies have tested cKit+ CPCs after MI. For example, Hong et al. performed intracoronary injection of cKit+ CPCs after MI in mice demonstrating improvement in left ventricle function despite poor engraftment [66]. By 5 weeks after treatment, <0.5% of injected cKit+ cells remained in the heart, suggesting the functional benefits stem primarily from paracrine signaling rather than formation of new myocardium. Indeed, lineage-tracing studies confirm that endogenous cKit+ cells generate only rare cardiomyocytes in vivo [67]. Similar findings have been reported for Sca1+/CD31− progenitor cells using acute MI rodent models, where cell delivery improved cardiac function and ventricular remodeling predominantly through paracrine-mediated neovascularization and support of native cardiomyocyte function [68]. Consistent with this, genetic deletion of Sca-1 in mice impairs post-MI repair, suggesting endogenous Sca-1 cells contribute to ischemic remodeling, although likely through supportive signaling [69]. While humans and large animals lack an exact Sca-1 analog, these studies underscore the mechanisms by which CPCs may ameliorate ischemic injury. Cardiac side population (SP) cells, identified by Hoechst dye efflux, share substantial functional overlap with Sca-1+ and cKit+ progenitors and are mobilized after myocardial infarction, contributing mainly to vascular lineages and modest functional recovery through analogous paracrine mechanisms rather than direct cardiomyocyte formation [70]. Collectively, these CPC subtypes, including sparse developmental progenitors such as isl1+ and Nkx2.5+ cells, highlight a conserved reparative paradigm characterized by indirect modulation of the ischemic microenvironment with minimal durable engraftment [71].

Translating CPC-based therapy to large animal models has provided critical insights under conditions that more closely mimic human myocardial ischemia. Most notably, Crisostomo and colleagues performed a preclinical safety and feasibility study of porcine CPCs in an ischemic-reperfusion MI pig model [72]. Intracoronary infusion of porcine CPCs one-week post-MI produced dose-dependent improvements in left ventricular EF and ventricular remodeling, reduced infarct size as reflected by a higher myocardial salvage index, and demonstrated safe myocardial homing without coronary obstruction [72]. With the understanding of the mechanistic similarities of CPC subtypes from small animal models, cardiosphere-derived cells (CDCs) have become extensively tested in large animal models, as they represent a mixed population of heart-derived progenitors expanded from cardiac explants [73]. Work by Marbán and colleagues has paved the path for evaluation of CDCs in swine models. An early study compared intramyocardial injection of autologous CDCs versus intact cardiospheres in swine with anteroseptal MI demonstrated not only the safety of injections but also the effectiveness in improving left ventricular function [74]. This was confirmed in a subsequent study on intracoronary delivery of CDCs after reperfusion in acute MI showing significant cardioprotective effects [75]. Most recently, their laboratory investigated three-vessel intracoronary infusion of CDCs in a swine hypertensive model of heart failure with preserved ejection fraction (HFpEF) and demonstrated improvement in left ventricular diastolic function and reduction in fibrosis [76]. Importantly, the following studies did not encounter safety issues regarding arrhythmias or coronary obstructions. Large animal studies of CPCs and CDCs corroborate rodent findings, demonstrating that both intramyocardial and intracoronary delivery are feasible and safe, with consistent reductions in scar burden and preservation of viable myocardium despite modest improvements in global function. These structurally favorable, paracrine-mediated effects, characterized by enhanced angiogenesis, reduced fibrosis, and minimal long-term engraftment, provided the mechanistic and safety rationale for subsequent evaluation of CPC-based therapies in human clinical trials.

Clinical evaluation of resident CPCs in ischemic heart disease has consistently emphasized safety with variable efficacy across trials. Early trials, including SCIPIO and CADUCEUS trials, demonstrated the feasibility and safety of intracoronary delivery, with SCIPIO reporting a 12.3% increase in left ventricular EF one year after infusion of autologous cKit+ CPCs, and CADUCEUS showing significant scar reduction and increase in viable myocardium despite minimal change in systolic function [77]. Importantly, neither study reported cell-related adverse events. Subsequent early phase trials have reinforced this safety profile, in which the CAREMI trials demonstrated that intracoronary delivery of allogeneic cKit+ CPCs 5–7 days after MI was feasible, minimally immunogenic, and free of major adverse events, although it did not produce significant improvements in infarct size or EF at one year [78]. Similarly, the ALLSTAR trial found that while off-the-shelf allogeneic CDCs intracoronary infusion did not reduce scar size in the subacute-to-chronic post-MI setting, they were well tolerated and produced modest effects on ventricular remodeling [79]. Taken together, these early clinical trials of variations in CPC therapies demonstrate strong procedural and immunological safety, but only modest and variable efficacy in improving post-MI cardiac structure and function.

**Table 1:** **Comparative Table of Cellular Therapy Modalities for Ischemic Heart Disease.** The following table describes the key features, evidence-based mechanisms of action, broad therapeutic goals, key sources for cell derivation, advantages and limitations, developments for bioengineering strategies, and clinical findings and development of four foundational cell-based therapies: (1) mesenchymal stem cells (MSCs), (2) induced pluripotent stem cells (iPSCs), and (3) resident cardiac progenitor cells (CPCs).

Feature/Attribute	Mesenchymal Stem Cells (MSCs) [34,35,36,37,38,39,40,41,42,43,44,45,46,47,48,49,50]	Induced Pluripotent Stem Cells (iPSCs) [51,52,53,54,55,56,57,58,59,60,61,62]	Cardiac Progenitor Cells (CPCs)/Cardiosphere-Derived Cells (CDCs) [63,64,65,66,67,68,69,70,71,72,73,74,75,76,,77,78,79]
**Primary Mechanism of Action**	Predominantly paracrine: pro-angiogenic, anti-inflammatory, anti-fibrotic	Similar paracrine activity; potency still under investigation	Paracrine recruitment of endogenous repair; cardiomyogenic potential debated
**Therapeutic Goals**	Preserve myocardium, reduce fibrosis, improve perfusion and function	Scalable, standardized therapy with improved expansion, replace scarred myocardium	Enhance endogenous cardiac repair signaling
**Source**	Bone marrow, adipose tissue, umbilical cord	Differentiated from iPSCs via lineage programs	Adult cardiac tissue or cardiospheres (historically cKit+ enrichment; however, multiple subtype populations)
**Advantages**	Low immunogenicity, strong immunomodulation, most clinically tested	Unlimited expansion, longer lifespan, faster proliferation, improved homogeneity	Initial endogenous homing potential, cardiac lineage proximity
**Challenges/Limitations**	Autologous decline with age/disease; low retention after delivery	May have reduced immunosuppressive capacity; biomarker validation needed; Immature electrophysiology → arrhythmia risk	Controversial cardiomyogenic capacity; limited reproducibility
**Bioengineering Strategies**	Hydrogels (fibrin, Alg-RGD), collagen scaffolds, ECM patches	CXCR4/mitochondrial enhancement; Engineered Heart Tissues (EHTs), bioreactors, conductive scaffolds	Focus shifted to EV/exosome paracrine therapeutics
**Key Clinical Findings**	Improved LVEF, perfusion, 6MWD in scaffold-assisted CABG; DREAM-HF reduced MACE in inflammatory phenotypes	Emerging preclinical/early translational evidence	Limited translation for overall cardiac functional benefits
**Stage of Development**	Most advanced clinical trial experience	Preclinical → early trials (including patch trials)	Preclinical → early phase trials

#### 3.2.4. Bioengineered Delivery/Future Directions

Translational success for all cellular therapies depends critically on overcoming the profound limitations of rapid cell wash-out, poor cell survival, and lack of functional integration within the ischemic myocardium [34]. Standard delivery methods, particularly the intracoronary infusion of various cell therapies discussed, have demonstrated modest and sometimes controversial clinical benefit [80]. This confirms that the efficacy of cell-based injection into the coronary vasculature is limited by low retention [44]. Bioengineered materials represent a promising solution to this delivery hurdle. Bioengineered materials, particularly injectable hydrogels, address major delivery limitations by providing a biocompatible 3D matrix that recapitulates the native extracellular matrix and microenvironment to ideally improve cell retention compared with direct cell injection [81]. For example, human umbilical cord-derived MSC-laden collagen hydrogel intramyocardial injection in patients with chronic IHD undergoing coronary artery bypass grafting (CABG) not only improve post-MI cell retention compared with cell treatment alone but also protect cells from ischemic stress and fibrosis while providing mechanical support that limits adverse remodeling [82]. This 2020 randomized trial of 114 patients showed that combining intramyocardial human umbilical cord-derived MSCs with a collagen scaffold significantly improved perfusion, left ventricular ejection fraction, and 6 min walk distance at 12 months versus CABG alone [82]. A separate clinical trial with early Phase I/II data using autologous bone marrow-derived MSCs delivered in a hydrogel via catheter-based intramyocardial injection showed improvements in left ventricular function and myocardial perfusion [83]. This tangible success highlights that the optimization of the bioengineering delivery system is often the primary determinant of functional efficacy, translating cell potency into measurable clinical success.

## 4. Extracellular Vesicles as Acellular Therapeutics

### 4.1. Biogenesis, Classification and Therapeutic Potential of Extracellular Vesicles

Extracellular vesicles (EVs) are a diverse group of cell-derived, lipid-bilayer encapsulated structures which serve as carriers of proteins, lipids, RNAs and other bioactive cargo, enabling intercellular communication in physiological and pathological states [84]. Specifically, the International Society for Extracellular Vesicles recommends the generic term “EVs” unless the origin (biogenesis pathway) is clearly defined, because many studies cannot reliably discriminate subclasses based on biogenesis alone [85]. From a functional and translational standpoint, EVs are appealing as acellular therapeutics due to their natural origin, ability to relay molecular signals, relatively low immunogenicity, and avoidance of potential complications associated with live cell transplantation (e.g., uncontrolled proliferation, differentiation, tumorigenicity, immune rejection) [84]. In the cardiovascular field, the interest in EVs is rapidly growing as a next-generation therapeutic modality that may harness the benefits of cell-based therapy while circumventing many of its drawbacks.

Within the broad family of EVs, there are three main subtypes: exosomes, microvesicles (also called microparticles or ectosomes), and apoptotic bodies [86]. Exosomes (typically 30 nm to 150 nm in diameter) derive from the endosomal system: inward budding of endosomal membranes forms intraluminal vesicles within multivesicular bodies (MVBs), and subsequent fusion of MVBs with the plasma membrane leads to release of exosomes into the extracellular space [86,87]. These vesicles often carry tetraspanins (CD9, CD63, CD81), Alix, tumor susceptibility gene 101 (TSG101), and endosomal-sorting complex required for transport (ESCRT) components [87]. Microvesicles, in contrast, originate by direct outward budding and fission of the plasma membrane, and generally range in size from about 200 nm to 2000 nm; they carry membrane proteins reflective of their plasma membrane origin and may bear phosphatidylserine on their surface [86,88]. Apoptotic bodies are the largest vesicles (about 1 μm to 10 μm) released during programmed cell death, and can contain fragments of nucleus, mitochondria, and cytoplasm [86,89]. Due to overlap in size, marker expression and cargo composition, it is difficult to strictly differentiate between these subtypes in many experimental settings, causing many translational studies to use the general term “EVs”.

From a clinical and translational standpoint, EVs offer a potentially more controlled and safer approach compared to live cells. They are structurally amenable to long-term storage (potentially via lyophilization or cryopreservation), are less prone to the uncontrolled biological behavior of live cells, can carry engineered therapeutic cargo, and are fundamentally suited for standardization and large-scale production [90,91,92]. At the same time, some key challenges with EVs include establishing robust, high-throughput scalable manufacture, ensuring rigorous potency assays, and confirming optimal biodistribution and retention in target tissues [93]. In the cardiovascular context, leveraging EVs as a therapy relies on using three broad mechanisms: neovascularization/arteriogenesis, anti-apoptotic/cytoprotective signaling, and metabolic/inflammatory reprogramming of injured myocardium and vascular tissue.

### 4.2. Mechanisms of Action of EV-Based Therapeutics

EV-based therapies are pleiotropic in nature, delivering a cargo of microRNAs (miRNAs), long non-coding RNAs, messenger RNAs, proteins, lipids and metabolites which can affect wide-ranging cellular changes in recipient tissues [84]. Their therapeutic potential in ischemic heart disease, myocardial injury, microvascular dysfunction and heart failure depends on three interconnected mechanisms: (1) angiogenesis and arteriogenesis, (2) anti-apoptotic and cytoprotective signaling, and (3) metabolic and inflammatory reprogramming.

#### 4.2.1. Promotion of Angiogenesis and Arteriogenesis

Human MSC-derived and CPC-derived EVs have repeatedly been shown to augment angiogenesis, in part by delivering pro-angiogenic miRNAs (including miR-126, miR-210, miR-21), growth factor mRNAs/proteins (e.g., VEGF, FGF, HGF), and modulating endothelial signaling pathways [16,94,95]. More specifically, human MSC-derived EVs with high levels of miR-210 downregulate the anti-angiogenic gene EFNA3 in endothelial cells, thereby unleashing pro-angiogenic signaling and enhancing capillary formation [96]. In a similar fashion, human MSC-derived exosomes with miR-132 markedly increase endothelial tube formation in vitro and enhance neovascularization in infarcted mice hearts, resulting in preserved cardiac function [97]. In addition, in rodent studies, MSC-derived exosomes overexpressing microRNA, miR-30e reduced lectin-like oxidized low-density lipoprotein receptor 1 (LOX-1) expression, down-regulated nuclear factor kappa beta (NF-κB) and caspase-9 signaling, reduced apoptosis and fibrosis, and improved function in myocardial infarct models, highlighting the mechanistic link between cytoprotection and angiogenesis [98]. These findings indicate that the angiogenic response in injured myocardium may be enhanced by EVs both by direct stimulation of endothelial/progenitor cells and by alteration of the injured microenvironment.

CPC-derived exosomes exhibit comparable pro-angiogenic properties: CPC-derived exosomes from adult Sprague-Dawley rat hearts secreted under hypoxic stress (incubated for 12 h under hypoxic gas mixture of 95% N_2_ and 5% CO_2_) contain upregulated clusters of angiogenic miRNA (e.g., miR-210) and have been shown to significantly enhance endothelial tube formation while concomitantly suppressing pro-fibrotic gene expression in the post-MI mouse heart [99]. These findings indicate that stem cell EV cargo (particularly microRNAs) can recapitulate the pro-angiogenic paracrine effects of their parent cells in ischemic tissue.

#### 4.2.2. Anti-Apoptotic Signaling and Cytoprotection

Beyond vessel-centric rescue, EVs may attenuate cardiomyocyte and stromal cell apoptosis directly, thereby preserving myocardial architecture and microvascular function. This is especially relevant in chronic ischemia, where maintenance of the microvasculature is essential for mitigating tissue loss and preventing remodeling. A recent review of EV-mediated cardiac repair emphasized that the benefits of EVs include reduction in cardiomyocyte apoptosis, modulation of endothelial and fibroblast survival, and attenuation of adverse remodeling [86].

EVs from both MSCs and CPCs blunt cardiomyocyte apoptosis and promote cell survival after infarction by delivering miRNAs, lncRNAs and proteins that regulate key survival pathways (including PI3K/AKT, Bcl-2 family, HSP70, HIF1α) [100]. A prime example is miR-21-5, one of the most abundant miRNAs in MSC secreted exosomes, which activates the PI3K/Akt pathway by silencing PTEN and other pro-apoptotic genes in recipient cardiomyocytes [101]. Furthermore, hypoxia-preconditioned (72 h cultured in 95% N_2_ and 5% CO_2_) MSC-derived EVs were further enriched in miR-125b-5p; this cargo inhibits the p53/BAK1 apoptotic axis in cardiac cells, as evidenced by loss-of-function experiments where knocking down miR-125b in MSC exosomes abrogated their ability to suppress p53/BAK1, leading to larger infarcts and reduced cardiomyocyte survival [102]. In similar studies, MSC-derived exosomes enriched with miR-22 demonstrated a reduction in apoptosis of cardiomyocytes by downregulating the Mecp2 gene [103].

After treatment with EVs secreted by adipose-derived MSCs in an acute MI mouse model, miR-671 expression was found to be increased and associated with a reduction in myocardial cell apoptosis and myocardial fibrosis by targeting the transforming growth factor beta receptor 2 and inhibiting SMAD2 phosphorylation [104]. From the same cellular source, exosomes derived from miR-146a-modified adipose-derived stem cells downregulated the early growth response factor 1 suppressing TLR4/NFκB signaling attenuating cell apoptosis and the inflammatory response [105].

Similarly, CPC-derived EVs convey cytoprotective miRNAs, for instance, miR-935 was identified as a highly expressed CPC-derived EV microRNA that mitigates oxidative stress-induced cardiomyocyte apoptosis, in which inhibition of miR-935 in these vesicles led to significantly increased apoptotic cell death [106]. Through a multitude of signals, multiple stem cell-derived EVs attenuate myocardial apoptosis and limit cellular damage due to ischemia.

#### 4.2.3. Metabolic Reprogramming and Modulation of Inflammation

In the context of IHD, injured myocardium and microvasculature are subject not only to hypoxia and nutrient deprivation but also to inflammatory activation, metabolic dysregulation (involving mitochondrial dysfunction, a shift to glycolysis, and increased reactive oxygen species) and microvascular rarefaction. EV-mediated modulation of metabolic and inflammatory pathways is thus a third critical mechanism for enabling cardioprotection. From a metabolic standpoint, EVs derived from MSCs, and other progenitor cells, have been shown in preclinical studies to enhance mitochondrial biogenesis, preserve mitochondrial membrane potential, shift metabolism from glycolysis toward oxidative phosphorylation, reduce lactate accumulation and reactive oxygen species (ROS) production, and thereby improve cellular energetic state and survival.

For example, under ischemic conditions, MSC-derived EVs transfer miR-210 and miR-21, which drive a shift from fatty acid oxidation toward glycolysis by upregulating GLUT1 transporters and pyruvate dehydrogenase kinase (PDK1), an adaptation that improves ATP production efficiency in the myocardium [107,108]. Another similar regulation involves MSC-derived exosomes carrying miR-100, which suppresses CD36 expression, a critical gene for oxidative phosphorylation, thereby reinforcing the notion of the metabolic shift from fatty acid oxidation to glycolysis in the ischemic myocardium [109]. Beyond the switching of metabolic substrates, EVs have been shown to regulate mitochondrial function and redox homeostasis [110]. Additional miRNAs such as miR-9, miR-29b, miR-320, miR-455, miR-599, and miR-122, have been found to profoundly impact and regulate mitochondrial energetics and biosynthesis. Specifically, miR-9 influences fission and fusion by targeting Drp1, meanwhile miR-29b enhances mitochondrial integrity by upregulating PGC-1α protein [111,112]. Furthermore, miR-320 and miR-455 upregulate superoxide dismutase (SOD) expression to help maintain redox balance [113,114].

In tandem with metabolic reprogramming, EV cargos exert significant immunomodulatory effects. Notably, both MSC-derived and CPC-derived EVs have been shown to carry anti-inflammatory microRNAs, including miR-125a-5p, miR-10a, miR-146a, and miR-21. Gao et al. demonstrated that miR-125a-5p modulates macrophage function shifting towards the anti-inflammatory M2 phenotype by targeting Klf13, Tgfbr1, and Daam1 after myocardial ischemia/reperfusion injury in both mice and swine [115]. Similarly, endothelial cell-derived EVs transfer miR-10a, which suppresses NFκB signaling where MSC-derived and CPC-derived EVs carry miR-146a that interfere with toll-like receptor signaling, thereby dampening NFκB-mediated inflammatory responses in the ischemic myocardium [116,117]. Likewise, MSC-derived EV delivery of miR-182-5p was shown to inhibit the pyroptosis executor protein GSDMD, reducing inflammasome-driven cell death and inflammatory cytokine release of ischemia/reperfusion injury in mice [118]. By simultaneously modulating cellular energy pathways and inflammatory signaling, MSC- and CPC-derived EVs foster a more regenerative, less hostile metabolic and immune environment in the ischemic heart. The combination of these mechanisms, including angiogenesis, survival signaling, and metabolic/inflammatory modulation, positions EVs as a promising therapeutic for the injured myocardium and microvascular dysfunction.

### 4.3. Translational Large Animal Evidence

Given the mechanistic literature in small animals, we now focus on large animal models describing the effects of EV-based therapies. Several important studies have investigated EV-based therapies in swine models of chronic myocardial ischemia, metabolic syndrome, or combined pathologies. These studies are particularly salient because they go beyond rodent studies and better reflect the clinical milieu of adult human cardiovascular disease.

One foundational swine study injected human MSC-derived EVs intramyocardially into Yorkshire swine after placement of an ameroid constrictor on the left circumflex artery to induce chronic ischemia [119]. Five weeks post-treatment, treated animals exhibited increased myocardial blood flow, improved ischemic/non-ischemic perfusion ratio, elevated capillary density, and higher alpha smooth muscle actin (α-SMA)-positive arterioles than controls. Furthermore, the researchers found that phospho-eNOS/eNOS and phospho-MAPK/MAPK were elevated, and AKT was upregulated. The authors concluded that MSC-EV injection enhances perfusion and micro-vascular structure in chronic ischemic myocardium via MAPK and AKT/eNOS signaling [119].

Subsequently, to investigate the effects of EV injection in a more complex disease environment, a study of EV injection in a swine model of metabolic syndrome and chronic ischemia found that EV treatment significantly decreased myocardial pro-inflammatory signaling compared with controls [120]. A companion study in that same model showed that human MSC-derived EV intramyocardial therapy lowered myocardial expression of anti-angiogenic proteins, including angiostatin, endostatin, and cathepsin D as well as demonstrated an inverse correlation between angiostatin expression and blood flow (r_s_ = −0.76; *p* = 0.037) [16]. These findings are particularly important because they indicate that EV therapies can overcome metabolic syndrome–induced resistance to neovascularization.

More recently, studies have used “hypoxia-conditioned” (24 h incubation in 5% carbon dioxide and 95% nitrogen) human bone marrow MSC-derived EVs in chronically ischemic swine myocardium showed increased expression of several antioxidant proteins and altered redox signaling, as well as a decrease in overall apoptosis through the activation of key pro-survival pathways (PI3K/Akt) [121,122]. Notably, a comparative study was performed examining the differences between normoxia serum-starved (for 24 h) human MSC-derived EVs and hypoxia-modified (same 24 h preconditioning as above) MSC-derived EVs in a swine model of chronic ischemic heart disease. This critical study demonstrated that hypoxia-modified EVs enhanced contractility, capillary density, and angiogenic signaling compared to normoxia serum-starved EVs [123].

Collectively, these swine studies demonstrate several critical translational insights: (a) intramyocardial delivery of EVs is feasible and safe in large animals; (b) EV therapy improves microvascular density, perfusion and structure in chronically ischemic myocardium; (c) EV therapy retains efficacy even in challenging comorbid conditions such as metabolic syndrome; (d) mechanistic links to angiogenesis, anti-inflammatory and anti-angiogenic signaling, metabolic/inflammatory reprogramming are present in large animals; and (e) donor cell preconditioning may further enhance effects. Despite these promising results, large-animal EV studies remain relatively few in number, and standardized dosing, controls, long-term follow-up, and functional outcomes remain to be uniformly reported. Nevertheless, these swine studies provide compelling evidence that EVs can produce meaningful structural and perfusion changes in clinically relevant models of chronic ischemia.

### 4.4. Clinical Trials and Current Human Translation of EV Therapy

The translation of EV therapeutics into human clinical trials is in its early phases, although there has been a greater focus on it in recent years. In particular, several observational and interventional trials have begun to evaluate the safety, feasibility, and efficacy of EVs in cardiovascular disease [124]. One pioneering human study, the SECRET-HF trial reported the first-in-man use of a cardiac progenitor cell-derived secretome, including EV-enriched fractions, in 12 patients with dilated cardiomyopathy or heart failure [125]. The trial was designed to evaluate safety and feasibility of repeated intravenous injections of the secretome. The authors reported no major adverse events, demonstrated reproducible EV/secretome product characteristics (via nanoparticle tracking analysis and other measures) and initial structural/functional cardiac data were promising [125]. The study supports the viability of EV/secretome therapies in human heart failure but underscores the need for larger randomized trials with functional endpoints.

In addition, a substudy of the SCIENCE trial, which randomized patients with ischemic heart failure to intramyocardial injection of adipose-derived stem cells (ADSC) vs. placebo, evaluated small EV miRNA expression in 15 patients at baseline and up to 12 months [126]. There was no significant change in small EV concentration post-treatment, but miR-126 expression declined significantly at 12 months in the ADSC group compared to the placebo, and circulating EV-miRNAs correlated with cardiac biomarkers. The authors concluded that this EV/miRNA data likely reflects upstream cellular effects of the ADSC therapy rather than direct EV therapeutic infusion; nonetheless, it emphasizes how EVs and their cargo may serve as biomarkers and may be modulated by interventional therapies [126].

The therapeutic potential of EVs extends beyond cardiovascular disease into diverse fields such as oncology and neurology. For instance, a critical review of all EV clinical trials highlighted the wide and growing scope of the field, noting that many therapeutic trials are currently underway, with a large segment focused on non-cardiovascular conditions [127]. While these new fields also face major challenges in manufacturing, dosing, and biodistribution, the increasing number of clinical trials involving EVs as a therapeutic mechanism confirms the broad therapeutic potential of extracellular vesicles across multiple disease categories.

### 4.5. Engineering Strategies to Enhance EV Yield, Targeting, and Potency

While native EV therapies show promise, their translation to the clinic demands further engineering refinements to overcome key limitations. Some limitations include low yield of therapeutic EVs from donor cells, heterogeneity of EV populations, inefficient targeting to injured myocardial tissue, rapid in vivo clearance due to sequestration in the liver or spleen, and modest potency in deliverable doses. Engineering strategies to address these challenges include donor cell preconditioning and EV yield enhancement, surface modification/targeting, cargo loading/engineering, delivery vehicles/biomaterials, and standardization/manufacturing scale.

#### 4.5.1. Donor Cell Preconditioning and Enhanced EV Yield

One route to improve EV quantity and quality is through donor-cell modulation. Preconditioning donor cells with hypoxia, shear-stress, cytokines, nutrient deprivation or pharmacologic agents can increase EV release and alter cargo towards more reparative phenotypes. For example, hypoxia preconditioning of MSCs increased exosome function in terms of myocardial repair and enhanced cardioprotection in a murine model of myocardial ischemia/reperfusion, as evidenced by improved cardiomyocyte survival [128]. In large-animal settings, use of hypoxia-conditioned (24 h incubation in 5% carbon dioxide and 95% nitrogen) human MSC-derived EVs in chronically ischemic swine myocardium increased expression of antioxidant defense proteins in recipient tissue [121]. From a manufacturing perspective, culture in 3D bioreactors, use of microcarrier systems, serum-free chemically defined media, and tangential flow filtration have been proposed to support scalable EV production [129].

In addition to preconditioning, direct genetic engineering of donor cells can bolster EV yield and consistency. For instance, Lai et al. demonstrated that immortalizing MSCs by c-Myc overexpression yielded a cell line capable of sustained, GMP-compliant EV production [130]. Such engineered cell lines of EV “biofactory” cells can be created via CRISPR/Cas9 to stably overexpress therapeutic cargo (e.g., pro-angiogenic microRNAs) or to display surface ligands on EVs, enabling the consistent production of “designer” EVs. Another approach is to derive EV-producing cells from expandable sources: induced pluripotent stem cell-derived MSC-like cells have shown higher EV output than primary MSCs [131], potentially overcoming the proliferation limits of donor cells. These cell engineering strategies can significantly increase EV yields and tailor EV content without continual donor harvesting.

#### 4.5.2. Targeting Enhancement and Surface Modification

Another major translational hurdle is that systemically delivered EVs are rapidly cleared by the mononuclear phagocyte system (MPS), with predominant accumulation in liver, spleen and lungs rather than heart tissue [132]. To circumvent this, several targeting strategies have been developed. These include (a) decoration of EV surfaces with cardiac-homing peptides (e.g., CSTSMLKAC) or antibodies directed at myocardium/endothelial receptors, (b) cloaking EVs with platelet- or leukocyte-derived membranes to evade MPS uptake and increase cardiac tropism, (c) incorporation of magnetic nanoparticles enabling magnetic guidance, (d) hydrogel encapsulation to localize EVs to the myocardium, and (e) two-step delivery schemes involving temporary macrophage inactivation followed by therapeutic EV delivery [133,134,135,136]. These modifications aim to increase EV retention in injured myocardium, reduce off-target uptake, and enable lower therapeutic doses.

Emerging physical targeting approaches also show promise. In magnetic targeting, EVs can be loaded or surface-tagged with iron-oxide nanoparticles, allowing magnetic fields to concentrate EVs at the heart and even permit MRI tracking of their delivery [137]. Wang and colleagues’ recent work demonstrated that magnetically labeled iPSC-derived EVs could be guided to infarcted myocardium under MRI/MPI, enhancing local retention and regenerative effects [138]. Likewise, acoustic guidance using ultrasound can improve EV delivery. Ultrasound-targeted microbubble destruction (UTMD) has been used to transiently open biological barriers and drive EVs into target tissues [139]. For example, focused ultrasound in combination with microbubbles was shown to increase EV concentration in a targeted region (e.g., across the blood–brain barrier) by cavitation-enhanced extravasation [139]. Such magnetic and acoustic targeting strategies complement biochemical modifications, offering externally controlled means to direct EVs to the ischemic myocardium and potentially improving therapeutic efficacy.

#### 4.5.3. Cargo Engineering and Functional Modulation

Beyond targeting and delivery, engineering of EV cargo offers powerful opportunities to upregulate therapeutic potency. Approaches include genetic modification of donor cells to overexpress specific miRNAs/lncRNAs or proteins; loading of isolated EVs via electroporation, sonication or extrusion; surface conjugation of aptamers or targeting ligands; and integration of small-molecule drugs inside EVs [140,141,142]. In the cardiovascular domain, one primate model study loaded miR-486-5p into EVs, which then outperformed unmodified EVs in terms of improving left ventricular ejection fraction and vascular density [143]. Such engineered EVs could potentially overcome the modest effects of naïve EVs, allowing for dose reduction and repeat dosing. More broadly, “designer” EVs can be programmed to carry cardioprotective RNAs or proteins that counteract ischemic injury pathways, or even genome-editing tools to address cardiac disease at the genetic level. These cargo-enhanced EVs could overcome the modest effects of unmodified EVs, providing greater functional improvement in models of myocardial infarction and heart failure.

#### 4.5.4. Biomaterial/Delivery Platforms and Sustained Release

Local retention of EVs in target myocardium is a critical challenge, as simple bolus injections often result in wash-out, diffusion away from the border zone, and limited cellular uptake [144]. To address this, the biomaterial platforms offer a compelling solution, with systems such as injectable hydrogels, conductive scaffolds, and patch systems having been successfully used to load EVs in preclinical models [145]. For instance, an injectable conductive hydrogel loaded with umbilical cord MSC-derived EVs improved cardiac function and reduced fibrosis in a rat myocardial infarct model; the biomaterial provided prolonged local EV release and increased cardiac retention [146]. Similarly, cardiac patches incorporating EVs or EV-producing cells can mimic extracellular matrix properties and deliver vesicles over time at the sire of injury. A recent study embedded EVs (from macrophage engineered with miR-199a-3p) into a 3D-bioprinted cardiac patch, which led to a threefold higher cardiomyocyte viability in the patch and enhanced cell proliferation compared to patches without EVs [147]. Such 3D-engineered patches illustrate how combining EV therapy with tissue engineering achieves spatial control of EV delivery and more durable therapeutic effects in vivo. Notably, 3D bioreactor cultures can dramatically increase EV yields, one study reported a ~5–8 fold increase in MSC EV output using microcarrier-based 3D culture versus 2D flasks, and up to 140-fold higher yields when combining bioreactors with tangential-flow filtration for isolation [148]. Ensuring the resulting EVs maintain their therapeutic potency is crucial: recent proteomic analyses indicate EVs from 3D cultures can retain similar or enhanced functional profiles relative to 2D culture EVs [149].

Beyond natural EVs, researchers are developing synthetic EV mimetics and biomimetic nanoparticles as alternative delivery vehicles. These are artificial nanoparticles designed to replicate the size, composition, or surface markers of natural exosomes [150]. By incorporating selected membrane proteins or coats from cells, biomimetic nanocarriers can fuse biological and synthetic components to improve targeting and stability [150]. For example, Bejerano et al. demonstrated that hyaluronan-sulfate nanoparticles loaded with a miR-21 mimic could home to infarcted myocardium, be taken up by cardiac macrophages, and induce their switch from pro-inflammatory to reparative phenotype [151]. Treated mice showed increased angiogenesis and reduced myocardial fibrosis and apoptosis, highlighting that an exosome-mimetic nanocomplex can effectively augment cardiac repair. Such biomimetic EV analogues offer advantages in manufacturing scalability and cargo loading, potentially addressing some limitations of native EVs. In parallel, cell-membrane-coated nanoparticles (using membranes from MSCs or CPCs) are being explored to confer natural homing capabilities and immunocompatibility to synthetic carriers [150]. Overall, these biomaterial and bioinspired platforms, ranging from hydrogels and scaffolds to exosome mimetics, enable more precise and sustained delivery of EV-associated therapeutic factors to the injured heart.

#### 4.5.5. Potency Assays, Standardization and Manufacturing Scale

For translation, it is imperative to establish standardized potency assays assessing metrics including endothelial cell migration, tube formation, recipient cardiomyocyte survival, and myocardial perfusion in vivo [93]. It is also important to define dosing metrics and implement rigorous quality control of EV preparations, in terms of size, concentration, surface markers, sterility, endotoxin, and stability [152]. A recent systematic review emphasized that many preclinical EV studies suffer from heterogeneity in isolation methods, dosing units, route and timing of administration, and outcome measures, hindering meta-analysis [153]. To improve preclinical predictive power, advanced organ-on-a-chip and ex vivo models are now being employed alongside traditional assays. For example, a microfluidic heart-on-a-chip platform was used to test EV therapeutic efficacy under biomimetic cardiac conditions: endothelial cell-derived EVs containing protective proteins were shown to rescue contractile function after induced ischemia–reperfusion injury in a human engineered cardiac tissue chip [154]. As these platforms mature, they could become valuable tools in comparing EV products and optimizing dosing prior to in vivo trials.

Furthermore, large-scale manufacturing of EVs must address scalability, purification, stability, and cost-effectiveness. Good Manufacturing Practice (GMP)-compliant production platforms are being actively developed, including closed-system hollow-fiber bioreactors, automated perfusion systems, and scalable filtration/chromatography methods [148]. For example, 3D bioreactor cultures may allow generation of liters of EV-conditioned medium under sterile, controlled conditions [148]. As production scales up, rigorous standardization is needed. Every step of the biomanufacturing process (culture conditions, purification, storage) should be validated to meet defined release criteria for identity, purity, and potency. Regulatory frameworks for EV therapeutics are evolving: EVs may be regulated as biologics, advanced therapy medicinal products or combination products depending on cargo and modifications [155]. This regulatory complexity mandates that demonstrating consistent, safe, and reproducible EV production is a prerequisite for late-phase clinical trials.

## 5. Extracellular Matrix (ECM) and Bioengineered Scaffolds

### 5.1. ECM Role After Ischemic Injury

After an acute injury or chronic ischemia, the heart’s limited regenerative capacity means that healing relies on an ECM-driven wound repair process. Ischemic injury triggers acute ECM proteolysis by matrix metalloproteases (MMPs), followed by robust deposition of collagen and other matrix proteins, culminating in a non-contractile fibrotic scar [156]. Beyond these biomechanical effects, the altered post-ischemic ECM actively influences cell signaling. Degradation fragments of the ECM and upregulated matricellular proteins, such as tenascin-C and periostin, can abnormally activate inflammatory and profibrotic pathways in infarct or border zone cells. Such maladaptive ECM-derived signals, together with increased tissue stiffness, drive adverse remodeling that compromises cardiac output and contributes to heart failure progression [157,158]. In ischemic and infarcted hearts, ECM fragments and specialized proteins further regulate inflammatory recruitment, fibroblast activation, angiogenesis, and the balance between reparative scarring and adverse remodeling [159]. Pressure overload triggers early activation of a matrix-synthetic program in cardiac fibroblasts, inducing myofibroblast conversion and stimulating the synthesis of both structural and matricellular ECM proteins. The resulting expansion of the cardiac ECM increases myocardial stiffness and promotes diastolic dysfunction, while cardiomyocytes, vascular cells, and immune cells are concurrently activated through mechanosensitive and neurohumoral pathways [157].

### 5.2. Non-Cell-Based ECM Biomaterials and Their Role in Regenerative Therapy

#### 5.2.1. Decellularized ECM Hydrogels and Injectable Matrices

**Figure 2:**
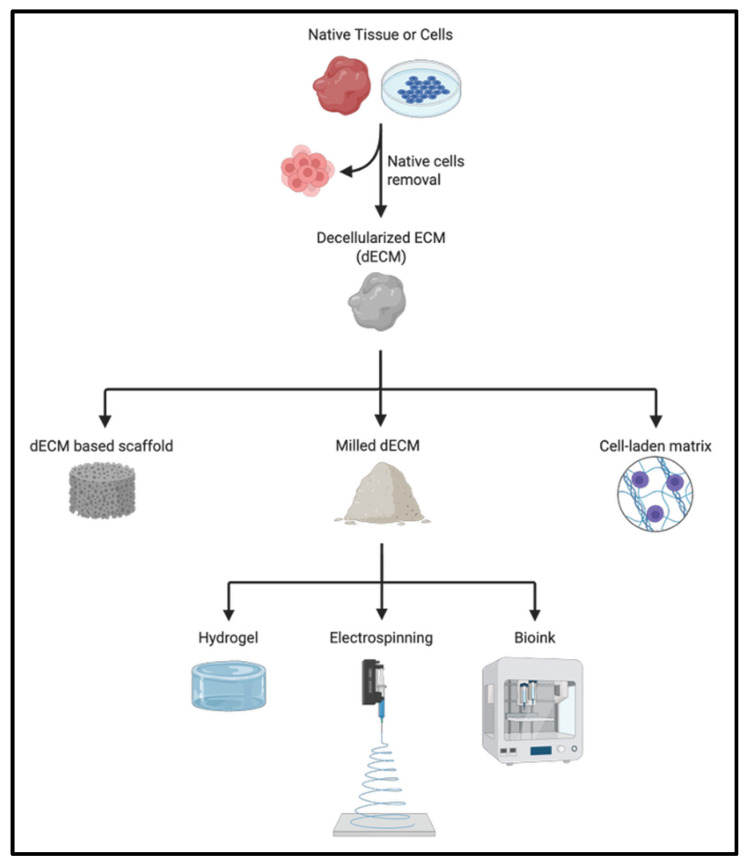
**Overview of Extracellular Matrix (ECM)-Based Biomaterials.** Schematic overview of the generation of ECM-derived therapeutic platforms. Native tissue or cell sources undergo decellularization to remove any cellular component (including DNA) meanwhile preserving the structural complexity of the ECM. The decellularized ECM (dECM) can then be processed into multiple forms: intact dECM scaffolds for direct implantation, milled dECM for reconstitution into hydrogels, electrospun fibers, or bioinks, and cell-laden matrices to combine cellular and acellular strategies.

Decellularized ECM (dECM)-based biomaterials represent a non-cell-based strategy designed to restore structural and biochemical cues lost after myocardial infarction (Figure 2). These scaffolds typically undergo mechanical or chemical processing to remove cells while retaining their native cardiac ECM components and architecture, providing structural support and biochemical cues to the injured heart [158]. Injectable hydrogels derived from porcine myocardial dECM have been extensively validated in rodent and swine MI models, where it promoted tissue-level regeneration; specifically, they were found to improve left ventricular function, increase viable myocardium, and reduce fibrotic scar formation relative to controls [160]. In another study both rodent and mini-swine ischemia–reperfusion models were used to evaluate a decellularized, intravascularly injectable ECM (iECM) therapy. In both species, the iECM selectively localized to leaky post-ischemic vasculature, reduced vascular permeability and myocardial edema, and maintained left ventricular structure and function without evidence of vascular obstruction. These consistent, cross-species outcomes demonstrate that acutely delivered iECM restores endothelial integrity and mitigates adverse remodeling, confirming translational conservation of its reparative mechanisms across small and large animal models [161]. Likewise, an “off-the-shelf” cardiac patch made from decellularized porcine myocardium (loaded with cell-derived growth factors but no living cells) dramatically improved post-MI recovery: treated rats had ~50% higher ejection fraction and ~30% smaller infarct scars than controls, and a pilot pig study confirmed reduced scarring and stabilized cardiac function [162].

Building on this, our lab developed a three-dimensional decellularized ECM particle injection derived from human umbilical cord MSCs in an effort to reduce immunogenicity and preserve native architecture. In a murine LAD-ligation model, intramyocardial (I/M) delivery of this ECM markedly reduced infarct size, improved contractility, and enhanced capillary density within the ischemic territory without provoking maladaptive inflammatory responses. Proteomic analysis indicated upregulation of contractile and metabolic pathways, suggesting enhanced tissue recovery and perfusion [163]. Furthermore, our laboratory is currently investigating this I/M ECM injection in a swine model of chronic myocardial ischemia to further evaluate its translational potential.

Distinct from injectable matrices, ECM-based epicardial patches provide localized mechanical support and sustained bioactive signaling at the infarct border zone [164]. The CorMatrix CorPatch, dECM patch made from pig small intestinal submucosa, has received FDA 501(k) clearance for epicardial infarct repair during coronary artery bypass grafting (CABG) surgery [165]. Case reports have demonstrated progressive myocardial scar shrinkage and increase in left ventricular function, suggesting the patch has adjunctive potential during current revascularization procedures [166]. Additionally, a PeriCord patch, which is obtained from allogeneic human pericardium, which was safely implanted in a patient after acute MI undergoing CABG without adverse events during the acute post-operative period along with ~9% reduction in scar mass after 3 months [167].

#### 5.2.2. Electrospun ECM

Another technique frequently explored is electrospinning (Figure 2). This involves a process in which nanofiber scaffolds are created by subjecting a polymer solution to a high-voltage field [168,169]. Not only is this process straightforward and scalable for patient use, it has also been shown in prior studies to preserve the mechanical, structural, and biological properties of cardiac ECM [168,170]. In rodent models, electrospun patches that incorporate ECM-like proteins (80% elastin, collagen, and polycaprolactone) have repeatedly shown anti-remodeling effects (with and without progenitor cell loading); post-MI treatment demonstrated improved cardiac function and reduced infarct area and LV remodeling over several weeks [171]. In parallel, electrospun ECM-derived scaffolds (e.g., decellularized porcine cardiac ECM-based patches) have been reported to moderate remodeling and scar formation with partial functional recovery in chronic rat MI models, supporting the concept that retaining ECM composition within an electrospun architecture can enhance “patch-only” efficacy (without loading cellular therapies) [168]. Lastly, another study in an acute infarction rat model demonstrated efficacy of an electrospun cardiac patch integrating decellularized pericardium. The treatment group exhibited significantly improved cardiac function, reduced left ventricular dilation and scarring, enhanced cardiomyogenesis, and promoted angiogenesis in the infarcted zone [172]. Taken together, these studies demonstrate how targeted matrix modulation can not only repair ischemic injury but also counteract the maladaptive structural and biochemical remodeling that defines post-MI pathology [173,174,175].

Large animal translation remains less mature for electrospun ECM specifically, representing an important knowledge gap. Similarly, human data are significantly limited—while some biomaterial cardiac patches have entered early clinical evaluation, dedicated post-MI trials of electrospun ECM products remain extremely sparse, making large animal studies an important bridge for defining dose/coverage, surgical handling, biodegradation, and safety endpoints that would support first-in-human translation.

#### 5.2.3. Synthetic-Natural Hybrid ECM Biomaterials

To improve tunability and manufacturability, hybrid ECM biomaterials combining natural ECM components (e.g., collagen, fibrin, dECM fragments) with synthetic polymers such as PEG, PLGA, and polycaprolactone (PCL) have emerged. These composites allow precise control over stiffness, degradation kinetics, and bioactive ligand density, enabling optimization for infarct mechanisms. Furthermore, these patches can be enhanced by loading drugs or cell-based therapies. In rodents, a PEG-fibrinogen hydrogel delivery VEGF improved infarct stabilization and neovascularization after MI, preserving left ventricular function and demonstrating how a hybrid matrix can couple mechanical support with sustained local bioactivity [176]. Similarly, electrospun PCL-collagen nanofibrous patches applied epicardially significantly reduced left ventricular remodeling and improved cardiac function in a rat MI model [171]. In a comparative study of biomaterials, Fu and colleagues determined that electrospun collagen/poly(l-lactic acid-co-ε-caprolactone) scaffolds created favorable neovascularization and mechanical support compared to gelatin/PCL scaffolds [177].

Translation-oriented studies further support the relevance of these hybrid systems. Engelmayr et al. developed anisotropic polycaprolactone scaffolds functionalized with ECM-derived proteins, demonstrating improved mechanical compliance and force transmission when applied to infarcted myocardium, findings that directly informed later large animal epicardial patch designs [178]. Other materials explored in clinically relevant models utilized intracoronary delivery of an in situ-forming alginate hydrogel in a swine ischemia–reperfusion model that demonstrated feasibility and improved left ventricular remodeling [179]. In humans, the PRESERVATION-1 trial tested intracoronary IK-5001 (an alginate hydrogel) after acute MI as a remodeling-prevention strategy; meanwhile the AUGMENT-HF trial evaluated Algisyl-LVR (an alginate hydrogel implant injected into the myocardium) in advanced heart failure and reported sustained 1-year improvements in functional capacity and clinical status [180,181]. Importantly, no completed human clinical trials have yet evaluated poly(l-lactic acid-co-ε-caprolactone/PCL-ECM hybrid patches for ischemic heart disease, which distinguishes these materials from alginate- or PEG-based injectable hybrids already tested clinically.

#### 5.2.4. Conductive or Mechanoresponsive ECM Scaffolds

To address impaired electrical coupling in ischemic myocardium, conductive ECM scaffolds incorporating materials such as graphene, carbon nanotubes, or gold nanowires have been explored to enhance electromechanical integration. In a neonatal rat model, carbon nanotube-incorporated gelatin methacrylate (GelMA) hydrogels significantly improved electrical signal propagation, enhanced cardiomyocyte synchronization, reduced scar formation, and improved left ventricular function compared to nonconductive controls [182]. Similarly, gold nanowire-embedded alginate scaffolds improved electrical coupling and contractile behavior of cardiomyocytes, highlighting the ability of conductive fillers to bridge nonconductive scar tissue and improve function integration [183].

Furthermore, dynamic or stiffness-adaptive ECM hydrogels have emerged to better match the evolving mechanical environment of the infarcted heart, where progressive stiffening drives maladaptive remodeling. These materials may undergo time- or stress-dependent changes in elasticity. For example, stress-responsive supramolecular hydrogels that stiffen under load but may soften during diastole theoretically reducing adverse remodeling and preserve ventricular compliance [184]. While large animal and human clinical trials specifically using conductive or mechanoresponsive ECM scaffolds remain limited, these developmental and small animal studies provide strong mechanistic proof that electrical conductivity and dynamic mechanical adaptability are powerful design principles for next-generation cardiac biomaterials.

### 5.3. Cell-Laden Scaffolds and Cell-Produced EV/ECM Hybrids

#### 5.3.1. ECM Scaffolds Combined with Stem or Progenitor Cells

In regard to cell-laden matrices, multimodal bioengineered therapies where the structural advantages of ECM-derived scaffolds are coupled with the biological activity of cellular therapies to achieve more durable and targeted myocardial repair (Figure 2). The biomaterial scaffolds are used to improve cell retention, spatial targeting, and paracrine potency in the ischemic myocardium, which addresses a major limitation of standalone cell injection. In small animal IHD models, several matrix + cell/progenitor designs have shown functional and structural benefit: for example, Bellamy et al. implanted a fibrin patch loaded with human embryonic stem cell (ESC)-derived cardiac progenitors (SSEA-1+) onto chronically infarcted rat hearts and observed sustained improvement in ejection fraction and LV remodeling with increased border-zone angiogenesis, despite no long-term donor cell persistence, supporting a predominantly paracrine mechanism [185]. Furthermore, Chung et al. demonstrated that an electrospun poly(l-lactic acid) (PLLA) scaffold loaded with VEGF and cardiac stem cells sustained the release of VEGF resulting in robust angiogenesis and cardiomyogenesis in acute myocardial infarction [186]. Likewise, another study in a rabbit model of chronic myocardial ischemia investigated a PLLA scaffold engineering with granulocyte colony-stimulating factor successfully integrated into the myocardium and exhibited stimulated neovascularization, reduced inflammation, modulated ECM remodeling, and ultimately improved cardiac function [187]. In parallel, clinically oriented “bioartificial myocardium” concepts have been explored using collagen matrices seeded with autologous bone marrow-derived cells to thicken infarct regions and reduce adverse remodeling signals in ischemic settings [188].

In humans with IHD/heart failure, matrix-assisted cell therapy has been feasible in early studies: Chachques et al. reported clinical feasibility of grafting a cell-seeded collagen matrix in ischemic cardiomyopathy, with the rationale of restoring ECM support while augmenting cellular repair signaling [188]. Similarly, ESCORT evaluated human embryonic stem cell-derived cardiovascular progenitors delivered on a fibrin patch in severe heart failure of ischemic etiology, demonstrating the technical feasibility of producing clinical-grade progenitors and acute safety, thereby establishing a translational pathway for scaffold-assisted progenitor delivery even as efficacy optimization remains an active need [189].

#### 5.3.2. ECM-EV Hybrid Biomaterials

Although not a cellular therapy, ECM-EV hybrid biomaterials extend the same “retention and targeting” logic to EVs as cell-laden scaffolds with the goal of preventing rapid EV clearance, enabling controlled release, and concentrating cardioprotective cargo within ischemic territories. For example, in a recent murine ischemia–reperfusion injury model, researchers developed an injectable gelatin methacryloyl (GelMA) hydrogel encapsulating miR-222–engineered, cardiac-targeted extracellular vesicles (TeEVs) to create a bioactive ECM–EV hybrid patch [190]. Pericardial injection formed an in situ hydrogel patch enabling controlled TeEV release and precise myocardial targeting. The Gel-TeEV composite significantly reduced infarct size, apoptosis, and inflammatory cytokine expression, while improving left-ventricular ejection fraction and limiting fibrosis at three weeks post-ischemic reperfusion injury. Mechanistically, the hybrid construct restored integrin-mediated cell–matrix, promoting more coordinated contractile function and myocardial compliance [190]. Lui et al. implanted an engineered hydrogel patch that slowly released EVs from iPSC-derived cardiomyocytes, reporting reduced cardiomyocyte apoptosis and infarct size with improved functional recovery in infarcted rat hearts, alongside EV enrichment for cardiac-relevant miRNAs [191]. These small animal studies support the concept that mechanically tunable matrices can amplify EV efficacy by sustaining local bioavailability. Further work has been performed to create an optimal hydrogel or patch to deliver the EVs; for example, Gomez-Cid and colleagues developed an optimized drug-delivery material with polyethylene glycol (PEG), cardiac ECM hydrogels and cardiac stem cell-derived EVs, which showed improved mechanical properties and on-site EV retention [192].

Large animal studies are beginning to test clinically realistic delivery routes and safety constraints: for example, in swine MI model, epicardial/pericardial administration approaches have been used to compare cardiosphere-derived cells or their EVs and demonstrate feasibility of local biologic delivery without the coronary-embolization concerns of some intracoronary approaches; however, did not demonstrate beneficial effect on cardiac function [193]. Human translation is still earlier for EV-matrix combinations specifically (most registered efforts are still defining EV safety/delivery as a biologic), but the trajectory established by scaffold-assisted progenitor/cell trials in IHD provides a clear regulatory and procedural template for how localized biomaterial delivery can be implemented surgically or percutaneously as EV products mature.

### 5.4. Clinical Translation of ECM-Based Therapies

To further investigate the translational potential of ECM-based therapies, a meta-analysis of 88 preclinical MI studies across mice, rats, and pigs demonstrated that ECM therapies consistently enhance cardiac recovery by improving ejection fraction, fractional shortening, stroke volume, and wall thickness while reducing infarct size. These benefits were consistent across species, models, and ECM types, with intramyocardial injection outperforming patch delivery. Although methodological rigor varied, the overall evidence supports that ECM therapy reliably improves post-MI structure and function, supporting its promise as a broadly applicable and translatable approach for cardiac repair [194]. Furthermore, another meta-analysis was conducted to investigate the efficacy of intracardiac hydrogel injections combined with cells, drugs, cytokines, EVs or nucleic acid therapies in preclinical models of acute MI [195]. This study reported improvements in heart function and morphology, with the greatest functional gains observed when EVs were incorporated. Together, these advancements highlight the emergence of ECM-integrated therapeutics as a clinically translatable strategy to not only stabilize damaged myocardium but also actively promote cardiac repair and remodeling.

## 6. Conclusions and Future Directions

While IHD continues to impose a substantial global burden, conventional revascularization strategies alone remain insufficient to restore microvascular integrity and prevent progressive myocardial loss. Across preclinical and clinical studies reviewed herein, bioengineered therapies, including stem and progenitor cells, extracellular vesicles, and ECM-based materials, have consistently demonstrated the capacity to preserve viable myocardium, promote angiogenesis, and modulate inflammatory and metabolic pathways, primarily through paracrine and microenvironment mechanisms rather than durable cellular engraftment. Small animal models have been indispensable for defining molecular mechanisms and therapeutic targets yet have often overestimated regenerative efficacy; in contrast, large animal systems, particularly swine models of MI and chronic ischemia, have revealed more modest but reproducible benefits centered on structural preservation, perfusion enhancement, and remodeling control, thereby providing a more realistic benchmark for clinical translation.

A central theme emerging from large animal and early human trials is that delivery strategy and tissue context are often more determinative of efficacy than the specific biologic payload itself. ECM scaffolds, hydrogels, epicardial patches, and engineered delivery platforms consistently enhance retention, spatial targeting, and durability of both cellular and acellular therapies, translating mechanistic promise into measurable physiological benefit. Similarly, EV-based therapeutics recapitulate many advantages of cell therapy while offering superior safety, scalability, and standardization potential, particularly when combined with biomaterials or engineered approaches that address biodistribution and potency. Importantly, clinical studies across cell-, EV-, and ECM-based platforms have repeatedly demonstrated procedural feasibility and favorable safety profiles, even as efficacy outcomes remain heterogeneous—underscoring the need for better alignment between therapeutic mechanism, disease stage, delivery route, and patient phenotype.

Looking forward, the convergence of advanced biomaterials, precise cell- and vesicle-based therapies, and integrative molecular analytics positions the field to overcome longstanding barriers in cardiac regeneration. The next generation of bioengineered therapies for IHD relies on optimizing both the therapeutic agents, such as the stem cell or EV, and the delivery matrix. Rather than pursuing cardiomyocyte replacement alone, the field is increasingly coalescing around therapies that stabilize the ischemic myocardium, restore microvascular function, and reprogram the hostile post-ischemic environment toward repair. Regenerative capacity and delivery efficiency can be improved through chemical and physical preconditioning, genetic modifications that improve cell survival, and scaffold-based support that directs cardiac-specific differentiation and provides essential mechanical cues. Moreover, bioengineered therapies involve highly specific molecular and cellular interactions within the myocardium, in which incorporating a precision medicine approach that accounts for sex-based differences and disease heterogeneity becomes crucial for developing efficient and targeted treatments. To expedite this bench-to-bedside journey, it is paramount to deploy highly predictive preclinical models, mitigate translational risks, and manage biological complexity. Future investments should focus on the continued refinement of large animal models, optimization of bioengineered platforms, and persistent enrollment in human clinical trials to ultimately bring regenerative therapies closer to a clinical reality for patients with ischemic heart disease.

## Data Availability

No new data were created or analyzed in this review. Data sharing is not applicable to this article.

## Abbreviations

The following abbreviations are used in this manuscript:

IHD:	ischemic heart disease
HFpEF:	heart failure with preserved ejection fraction
EV:	extracellular vesicle
GDMT:	guideline-directed medical therapy
MI:	myocardial infarction
ECM:	extracellular matrix
iECM:	injectable ECM
LCx:	left circumflex coronary artery
LAD:	left anterior descending coronary artery
MSC:	mesenchymal stem cell
iPSC:	induced pluripotent stem cell
CPC:	cardiac progenitor cell
HLA:	human leukocyte antigen
CDC:	cardiosphere-derived cell
CABG:	coronary artery bypass grafting
VEGF:	vascular endothelial growth factor
HGF:	hepatocyte growth factor
IGF-1:	insulin-like growth factor 1
MACE:	major adverse cardiac events
CXCR4:	C-X-C chemokine receptor type 4
MVBs:	multivesicular bodies
TSG101:	tumor susceptibility gene 101
TGFBR1	tumor growth factor receptor 1
PDK1:	pyruvate dehydrogenase kinase
PCL:	polycaprolactone
ESCRT:	endosomal-sorting complex required for transport
eNOS:	endothelial nitric oxide synthase
MAPK:	mitogen activated protein kinase
Akt:	protein kinase B
LOX-1:	lectin-like oxidized low-density lipoprotein receptor 1
NF-κB:	nuclear factor kappa beta
IL-1β:	interleukin 1 beta
CD11:	cluster of differentiation 11
ROS:	reactive oxygen species
α-SMA:	alpha smooth muscle actin
ADSC:	adipose-derived stem cells
MPS:	mononuclear phagocyte system
MMPs:	matrix metalloproteases
I/M:	intramyocardial
GelMA:	injectable gelatin methacryloyl
TeEVs:	cardiac-targeted extracellular vesicles
PLLA:	poly(l-lactic acid)
ANKRD1:	ankyrin repeat domain 1
NPPB:	natriuretic peptide B
CCR2:	C-C chemokine receptor 2
CD74:	cluster of differentiation 74
TPRV4:	transient receptor potential cation channel subfamily V member 4
